# A Rare Case of Neurosarcoidosis Overlapped with Sjogren’s Syndrome

**DOI:** 10.3390/jcm11195709

**Published:** 2022-09-27

**Authors:** Wenxin Cai, Ru Li, Jing He, Miao Shao, Zhanguo Li

**Affiliations:** Department of Rheumatology and Immunology, Peking University People’s Hospital, No. 11 Xizhimen South Street, Xicheng, Beijing 100044, China

**Keywords:** neurosarcoidosis, Sjogren’s syndrome, rituximab

## Abstract

The coexisting of sarcoidosis and Sjögren’s syndrome (SS) has long been neglected since sarcoidosis is considered as an exclusion criterion for SS. We described a 55-year-old woman, who was diagnosed with coexisting neurosarcoidosis and Sjögren’s syndrome for 16 years. She presented with erythema nodosum, progressive sensory and motor impairment of the extremities, dry mouth, and dry eyes. High-resolution computed tomography (HRCT) of the chest showed symmetrical pulmonary micronodules, interstitial changes, and enlarged mediastinal lymph nodes. Spine magnetic resonance imaging (MRI) showed syringomyelia and thickening of the T3-9 spinal cord. She was with positive ANA and anti-SSA antibodies, impaired function of the lacrimal, salivary gland and renal tubules. Biopsy of skin and lung nodules revealed non-caseous granuloma. Salivary gland biopsy showed focal lymphocyte infiltration. Classification criteria for sarcoidosis and Sjogren’s syndrome were fulfilled in this patient based on clinical and laboratory features. This case extends our understanding of overlapped Sjogren’s syndrome with sarcoidosis and provides a referential value for clinical diagnosis.

## 1. Introduction

Sarcoidosis and primary Sjögren’s syndrome (pSS) are both autoimmune diseases involving multiple organs, which have some similar clinical features. Sjögren’s syndrome is a chronic disease that mainly affects the function of salivary glands, lacrimal glands and other important organs. Sarcoidosis is characterized by non-caseating granulomatous inflammation [1]. Neurologic involvement of sarcoidosis—neurosarcoidosis (NS)—is rare [2]. Typical imaging performance of spinal cord neurosarcoidosis includes nodular and linear enhancement with T1 hypointense/T2 hyperintense. The gold standard for the diagnosis of NS is biopsy confirmation in the nervous system, which would show non-caseating granuloma. However, it is difficult to obtain specimens of the central nervous system. Pulmonary and mediastinal/hilar lymph nodes biopsies can also be used as a powerful diagnostic basis [2]. According to both the 2002 and 2016 ACR/EULAR classification criteria for Sjögren’s syndrome [3,4], sarcoidosis should be excluded before diagnosis. There are few reports of sarcoidosis coexisting with pSS [5]. Here, we report the coexistence of sarcoidosis and pSS in a 55 year old female involving the central nervous system.

## 2. Case Presentation

A 55 year old female had intermittent paresthesia in the right upper limb for 16 years and progressive numbness/tingling in the lower extremities for 4 years. She gradually developed difficulty in walking, and magnetic resonance imaging (MRI) of the cervical spine showed syringomyelia. She underwent 3 operations of “filum terminale resection”, “herniated cerebellar tonsil resection” and “suboccipital decompression”. There was a transient improvement after surgery. However, it did not stop the disease from progressing.

**Figure 1:**
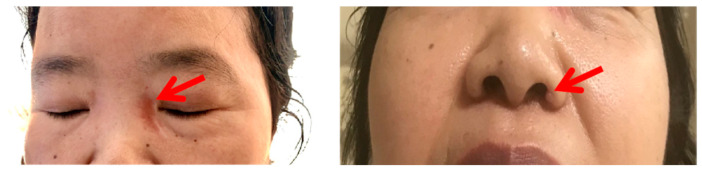
The erythema at the left inside canthus skin and the small nodule on the left nasal alar.

**Figure 2:**
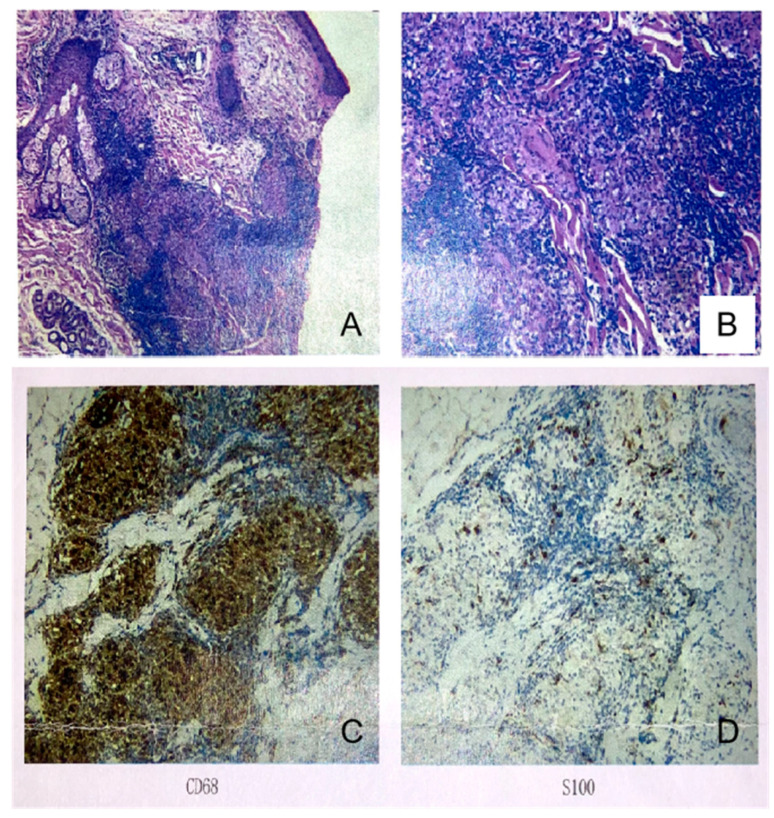
Left inside canthus skin biopsy stained with HE. (**A**) Atrophy and thinning of the epidermis. (**B**) Histiocytic proliferation and multiple nodules surrounded by scattered lymphocytic infiltration in the dermis. Histiocytes negative for CD1a, positive for (**C**) CD68, and scattered positive for (**D**) S100.

As her symptoms worsened, she was admitted to the Department of Rheumatology and Immunology in Peking University People’s Hospital. A detailed medical history was collected along with with careful physical examination. Skin lesions of the left inside canthus and nasal alar were noticed (Figure 1). The rash around the root nodules was about 3 × 1 cm in size, with no desquamation, no itching and no pain. The muscle strength of the left lower extremity was grade IV, and the muscle strength of the right lower extremity was grade II. The skin biopsy revealed non-caseating granulomatous (Figure 2). In addition, the patient had dry mouth and dry eye over the years, with a history of hypertension and Hashimoto’s thyroiditis.

**Figure 3:**
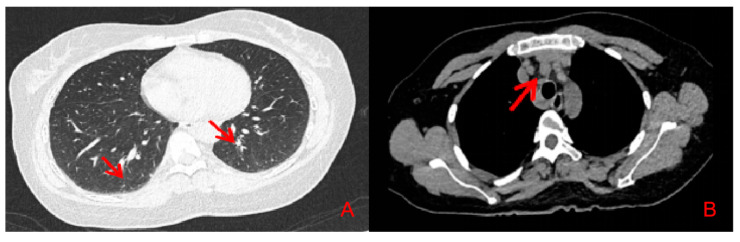
Chest CT shows symmetric (**A**) interstitial lesions closed to pleura, scattered micronodules in both lungs along with the bronchovascular bundles, and (**B**) multiple mediastinal enlarged lymph nodes.

**Figure 4:**
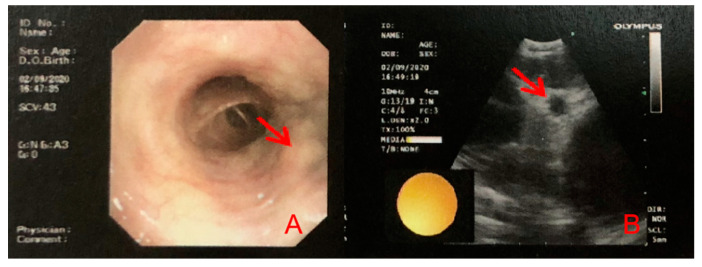
(**A**) Bronchoscopy shows multiple small nodules in the mucosa of the distal left main bronchus and the right middle bronchus. (**B**) Endobronchial ultrasonography shows enlargement of lymph nodes in 4L, 4R, 7, 11L, and 11R groups.

**Figure 5:**
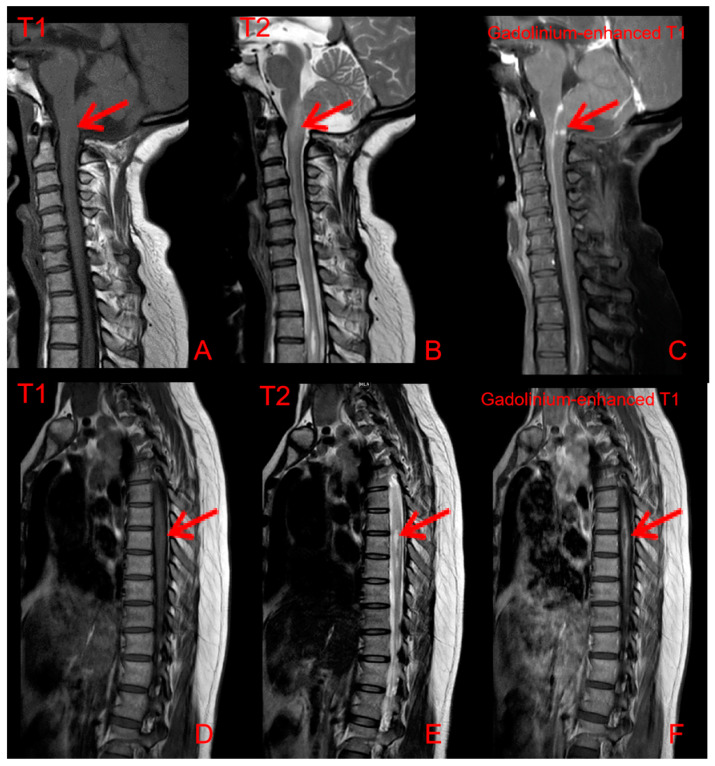
MRI of the spinal cord shows (**A**) hypointensity T1 signal and (**B**) hyperintensity T2 strips lesions below the level of the medulla oblongata, with slight thickening and swelling of the spinal cord. (**C**) Gadolinium-enhanced T1 MRI shows the line-like and nodular enhancement lesions at the level of C1 (SE 601A; IM 6), about 3 mm. (**D**) T1 phase, (**E**) T2 phase, and (**F**) gadolinium-enhanced T1 MRI shows the syringomyelia at the T3-4 level.

Her routine laboratory tests of complete blood count, renal and hepatic function, erythrocyte sedimentation rate (ESR), C-reactive protein (CRP) immunoglobulin and complement level were normal. Autoantibody spectrum showed that ANA (1:160), anti-Ro/SSA antibodies were all positive. The ANCA, and other inflammatory autoimmune disease-associated antibodies, was negative. Serum angiotensin-converting enzyme (SACE) did not increase (3.4 U/L, normal 17–55 U/L). HRCT showed symmetric pulmonary interstitial changes closed to pleura, micronodules distributed along with the bronchovascular bundles and multiple enlarged lymph nodes in the mediastinum (Figure 3). Bronchoscopy showed that there were many small nodules under the bronchial mucosa (Figure 4). The percentage of lymphocytes (23%) increased in bronchoalveolar lavage fluid (BALF), and the CD 4+/CD 8+ lymphocytes ratio was 11.46, which increased significantly. The histopathology of mediastinal lymph nodes in both group 7 and group 4R biopsies was non-caseating granulomatous. MRI showed abnormal signal and thickening of the spinal cord from the level of the medulla oblongata to the level of the spinal cord, with linear enhancement in the anterior part of the spinal cord, nodular enhancement in the posterior part of the spinal cord at the level of C1, and spinal cord cavity at the level of T3-4 (Figure 5). The lumbar puncture showed nonspecific immune-related inflammatory responses (Table 1). Testing of serum and CSF for infection (especially tuberculosis), asthenic bulbar paralysis, autoimmune encephalitis, multiple sclerosis, and Aquaporin 4 (AQP4, which is associated with neuromyelitis optica in Sjögren’s syndrome) were all negative. In addition, she was also examined for the function of the lacrimal gland (eye break-up time (BUT), Schirmer test) and salivary gland (salivary gland nuclear imaging), which showed severe impairment. Ultrasonographic examination of salivary glands revealed bilateral fibrosis changes. The labial glandular biopsy confirmed a focal lymphocytic sialadenitis.

She was diagnosed with sarcoidosis affecting the central nervous system and coexisting primary Sjögren’s syndrome. She received intravenous methylprednisolone pulse 500 mg daily for 3 days; the rash and paresthesia improved remarkably. Furthermore, the muscle strength of the right lower limb increased from grade 2 to grade 3. Then she was treated with cyclophosphamide (CTX) 400 mg every 2 weeks for 3 months, but the disease relapsed when glucocorticoids gradually were reduced to 25 mg per day. To control the disease, rituximab 500 mg was administered intravenously every week for 4 weeks. Three months later, she was able to walk 500 m with a walking aid and the dose of prednisone was reduced to 15 mg per day.

## 3. Discussion

Sarcoidosis is an idiopathic granulomatous disease affecting multiple organs, with the pathogenesis that the CD4+ T cells interact with antigen-presenting cells to initiate the formation and maintenance of granuloma [1]. In this case, the patient presented with erythema nodosum of skin, multiple lung nodules, enlargement of mediastinal lymph node, and elevation of lymphocytes, especially CD4+T cells, in bronchoalveolar lavage fluid. The biopsy specimens showed non-caseous granuloma in both erythema nodosum lesions and mediastinal lymph nodes. Therefore, the diagnosis of sarcoidosis was made.

Primary Sjögren’s syndrome is a multisystem disorder that shares certain clinical features with sarcoidosis. According to the 2002 and 2016 ACR/EULAR classification criteria for primary Sjögren’s syndrome, sarcoidosis should be excluded before the diagnosis of pSS [3,4]. Few studies have paid attention to the coexisting of sarcoidosis and Sjögren’s syndrome [5,6,7]. Casals et al., found that there is a higher prevalence of articular and ocular involvement, antinuclear antibodies (ANA), RF, and positive anti-Ro/SSA antibodies in patients with coexisting sarcoidosis and SS [5]. Although ANA could be detected in sarcoidosis, anti-Ro/SSA antibodies were uncommon. Due to the symptoms of dry mouth, dry eye, and the laboratory finding with the positivity of anti-Ro/SSA antibodies in these patients, Sjögren’s syndrome was suspected. A labial gland biopsy was made for this patient, which showed focal lymphocyte infiltration. However, the histologic data of exocrine gland biopsy for sarcoidosis is non-caseating granulomas. The diagnosis of Sjögren’s syndrome was made. Our case indicated that sarcoidosis and Sjögren’s syndrome can be diagnosed simultaneously. Analysis of immunologic profiles such as anti-Ro/SSA antibodies and histopathologic findings may be very useful. In sarcoidosis patients with positive anti-SSA/SSB antibodies and focal lymphocyte infiltration in a labial gland biopsy, specimens may suggest a coexistence with Sjögren’s syndrome.

The myelopathy of this patient was regarded as most likely to be sarcoidosis by non-invasive investigations, even though the biopsy is still the gold standard approach. When the spinal cord is involved, MRI of neurosarcoidosis often demonstrates T1 hypointense/T2 hyperintense lesions that are asymmetric and have nodular post-contrast enhancement and may develop into syringomyelia [8]. However, SS may present with longitudinally extensive transverse myelitis with optic nerve injury (neuromyelitis optica) [8,9]. In addition, AQP4-Ab in CSF, a sensitive and highly specific serum marker of neuromyelitis optica, may suggest SS. 

As some case reports and reviews [2,10,11] showed, rituximab and infliximab were both potential options to treat NS. Considering that the patient combined NS with SS, rituximab was thought to be a more optional drug to treat both diseases. As the symptoms improved after the rituximab therapy, we think that rituximab could be a potential option for NS overlapped with SS.

## 4. Conclusions

This rare case revealed that sarcoidosis and Sjögren’s syndrome can coexist simultaneously. It extends our understanding of the overlapped disorder and provides a referential value for clinical diagnosis.

## Figures and Tables

**Table 1:** Data of neuroimmune parameters.

Subject	Results	Reference Ranges	Meanings of Results
CSF OCB	Positive		Different from serum, type II
Serum OCB	Negative		
BBB permeability	21.01 × 10^−3^	<5.0 × 10^−3^	↑
IgG index	0.99	<0.85	↑
IgG-Syn (mg/24 h)	50.27	<7.0	↑
CSF MBP (µg/L)	1.92	<3.5	
Serum MBP (µg/L)	9.47	<2.5	↑
VSF MBP.Ab	0.296	<0.650	
Serum MBP.Ab	1.517	<0.750	↑
CSF MOG.Ab	0.337	<0.560	
Serum MOG.Ab	0.413	<0.640	
CSF NSE (µg/L)	12.44	<11.0	↑
Serum NSE (µg/L)	8.67	<9.0	
CSF S100β (µg/L)	1.68	<0.45	↑
Serum S100β (µg/L)	10.00	<0.35	↑

CSF, cerebrospinal fluid; OBC, oligoclonal bands; BBB, blood–brain barrier; immunoglobulin G; IgG-Syn, IgG-intrathecal synthesis rate; MBP, myelin basic protein; Ab, antibody; MOG, myelin oligodendrocyte glycoprotein receptor; NSE, neuron-specific enolase; S100β, central nervous system-specific protein; ↑: positive.

## Data Availability

Not applicable.
